# Roles of Organic Acid Anion Secretion in Aluminium Tolerance of Higher Plants

**DOI:** 10.1155/2013/173682

**Published:** 2012-12-27

**Authors:** Lin-Tong Yang, Yi-Ping Qi, Huan-Xin Jiang, Li-Song Chen

**Affiliations:** ^1^Department of Agricultural Resources and Environmental Sciences, College of Resources and Environmental Sciences, Fujian Agriculture and Forestry University, Fuzhou 350002, China; ^2^Institute of Horticultural Plant Physiology, Biochemistry, and Molecular Biology, Fujian Agriculture and Forestry University, Fuzhou 350002, China; ^3^Institute of Materia Medica, Fujian Academy of Medical Sciences, Fuzhou 350001, China; ^4^Department of Life Sciences, College of Life Sciences, Fujian Agriculture and Forestry University, Fuzhou 350002, China; ^5^Department of Horticulture, College of Horticulture, Fujian Agriculture and Forestry University, Fuzhou 350002, China

## Abstract

Approximately 30% of the world's total land area and over 50% of the world's potential arable lands are acidic. Furthermore, the acidity of the soils is gradually increasing as a result of the environmental problems including some farming practices and acid rain. At mildly acidic or neutral soils, aluminium(Al) occurs primarily as insoluble deposits and is essentially biologically inactive. However, in many acidic soils throughout the tropics and subtropics, Al toxicity is a major factor limiting crop productivity. The Al-induced secretion of organic acid (OA) anions, mainly citrate, oxalate, and malate, from roots is the best documented mechanism of Al tolerance in higher plants. Increasing evidence shows that the Al-induced secretion of OA anions may be related to the following several factors, including (*a*) anion channels or transporters, (*b*) internal concentrations of OA anions in plant tissues, (*d*) temperature, (*e*) root plasma membrane (PM) H^+^-ATPase, (*f*) magnesium (Mg), and (*e*) phosphorus (P). Genetically modified plants and cells with higher Al tolerance by overexpressing genes for the secretion and the biosynthesis of OA anions have been obtained. In addition, some aspects needed to be further studied are also discussed.

## 1. Introduction

Approximately 30% of the world's total land area and over 50% of the world's potential arable lands are acidic [1]. Furthermore, the acidity of the soils is gradually increasing as a result of the environmental problems including some farming practices and acid rain. Soil pH decreased significantly from the 1980s to the 2000s in the major Chinese crop-production areas [2]. Aluminium (Al) is the most abundant metal and the third most abundant element in the earth's crust after oxygen (O) and silicon (Si), comprising approximately 7% of its mass [3]. At mildly acidic or neutral soils, it occurs primarily as insoluble deposits and is essentially biologically inactive. In acidic solutions (pH < 5.0), Al becomes soluble and available to plants in the Al^3+^ and Al(OH)^2+^ forms [4]. Micromolar concentration of Al^3+^ can rapidly inhibit root growth. The subsequent impairments on water and nutrient uptake lead to poor growth and productivity [5]. Therefore, in many acidic soils throughout the tropics and subtropics, Al toxicity is a major factor limiting crop productivity [6]. Many plants have evolved different mechanisms for detoxifying Al externally, including secretion of Al-chelating substances (e.g., organic acid (OA) anions, phosphate (Pi), and phenolic compounds) from the roots, increased pH in the rhizosphere, modified cell wall, redistribution of Al, and efflux of Al [6–9]. Increasing evidence shows that the Al-induced secretion of OA anions from roots is a major mechanism leading to Al tolerance in higher plants [6, 9–15]. In this paper, we review the roles of the Al-induced secretion of OA anions from roots in Al tolerance of higher plants.

## 2. Aluminium-Induced Secretion of Organic Acid Anions from Roots

The Al-induced secretion of OA anions, mainly citrate, oxalate, and malate, from roots is the best documented mechanism of Al tolerance in higher plants. Plants differ in the species of OA anions secreted, secretion patterns, temperature sensitivity, response to inhibitors, and dose response to Al (see [9, 13], Table 1). Since the first report on the Al-induced secretion of malate from wheat (*Triticum aestivum*) roots [16], increasing evidence shows that many Al-tolerant species or cultivars are able to secrete high levels of citrate, malate, and/or oxalate from roots when exposed to Al, including barley (*Hordeum vulgare*) [17], maize (*Zea mays*) [18], buckwheat (*Fagopyrum esculentum*) [19, 20], rye (*Secale cereale*) [21], soybean (*Glycine max*) [22, 23], *Citrus junos* [24], sorghum (*Sorghum bicolor*) [25], triticale(×* Triticosecale *Wittmark) [26], *Polygonum* spp. [27], *Paraserianthes falcataria* [28], *Lespedeza bicolor* [29], *Citrus grandis*, and *Citrus sinensis* [30, 31]. All these OA anions (citrate, oxalate, and malate) secreted from plant roots can form stable, nontoxic complexes with Al in the rhizosphere, thereby preventing the binding of Al to cellular components, resulting in detoxification of Al [9, 12]. Of the three OA anions, citrate has the highest chelating activity for Al followed by oxalate and malate [9]. The Al-induced secretion of OA anions is localized to the root apex, which is in agreement with the targeting site for Al toxicity [20, 32, 33] and their secretion is highly specific to Al, neither phosphorus (P) deficiency nor other polyvalent cations result in the secretion of OA anions [20, 30, 34–38]. Based on the timing of secretion, two patterns of Al-induced OA anion secretion have been proposed [10, 11]. In Pattern I plants, no discernible delay is observed between the addition of Al and the onset of OA anion secretion such as buckwheat [39], tobacco (*Nicotiana tabacum*) [40], and wheat [37]. In this case, Al may simply activate a transporter in the plasma membrane (PM) to initiate OA anion secretion, and the induction of genes is not required [9, 11]. In Pattern II plants, OA anion secretion is delayed for several hours after exposure to Al such as in rye [22], *Cassia tora* [39], *C. junos* [24], soybean [41], *L. bicolor* [29], and triticale [26]. In this case, Al may induce the expression of genes and the synthesis of proteins involved in OA metabolism or in the transport of OA anions [12]. Yang et al. investigated the effects of a protein-synthesis inhibitor (cycloheximide, CHM) on the Al-induced secretion of OA anions from the roots of buckwheat, a typical Pattern I plant, and *C. tora*, a typical Pattern II plants, suggesting that both *de novo* synthesis and activation of an anion channel are needed for the Al-activated secretion of citrate in *C. tora*, but in buckwheat the PM protein responsible for oxalate secretion preexisted [39]. Although the Al-induced secretion of OA anions has been well documented, there is a lack of correlation between OA anion secretion and Al tolerance in some plant species. For example, the Al-induced secretion of OA anions (citrate and oxalate) cannot account for the genotypic differences in Al tolerance in maize, soybean, and buckwheat cultivars [42–44]. Wenzl et al. observed that the secretion of OA anions from Al-treated signalgrass (*Brachiaria decumbens*) apices was three- to 30-times smaller than that from Al-treated apices of buckwheat, maize, and wheat (all much more sensitive to Al than signalgrass) [45]. Ishikawa et al. investigated the amount of malate and citrate in Al media of seven plant species (Al tolerance order: *Brachiaria brizantha*, rice (*Oryza sativa*), and tea (*Camellia sinensis*) > maize > pea (*Pisum sativum*) and *C. tora* > barley) and of two cultivars with differential Al tolerance each in five plant species (rice, maize, wheat, pea, and sorghum). They did not observe any correlation of Al tolerance among some plant species or between two cultivars in some plant species with the amount of citrate and malate in Al media [46]. Yang et al. showed that eight oxalate accumulator cultivars from four species including* Amaranthus* spp., buckwheat, spinach (*Spinacia oleracea*), and tomato (*Lycopersicon esculentum*) secreted oxalate rapidly under Al stress, but oxalate secretion was not related to their Al tolerance [47]. Therefore, it is reasonable to assume that some plant species may contain other (stronger) mechanisms, which mask the effect of OA anions and/or that the Al-induced secretion of OA anions is too low to be an effective mechanism [44, 48–50]. In this section, we will discuss several aspects that have been implicated in the regulation of the Al-induced OA anion secretion.

### 2.1. Anion Channels or Transporters

From the experiments with anion channel and carrier inhibitors, the Al-activated secretion of OA anions is mediated through anion channels and/or carriers [9, 20, 37, 69]. As early as 1995, Ryan et al. observed that inhibitors of anion channels inhibited the Al-activated secretion of malate from wheat roots, providing evidence that Al might activate malate secretion *via* a channel in the PM in the apical cells of Al-tolerant wheat cells [37]. Increasing evidence shows that the influence of anion channel inhibitors on the Al-activated secretion of OA anions depends on the species of OA anions secreted, plant species, inhibitor concentration, and species (see [13, 30, 37, 62], Table 1). Li et al. observed that two citrate carrier inhibitors (pyridoxal 5′-phosphate (PP) and phenylisothiocyanate (PITC)) effectively inhibited citrate secretion, meaning that the Al-activated citrate from rye roots is mediated by citrate carrier [21]. Yang et al. [36] and Li et al. [63] showed that the Al-activated secretion of citrate from rice bean (*Vigna umbellata*) and *Stylosanthes* spp. roots was inhibited by both anion channel and carrier inhibitors, indicating the possible involvement of both the citrate carrier and anion channel in the Al-activated citrate secretion. Although the use of inhibitors can be indicative of the type of transport protein involved in OA anion secretion, they do not provide definitive evidence because most inhibitors will eventually affect transport processes that can happen nonspecifically depending on the concentration and period of application. The use of patch clamp technique, which directly measures the transport activity, provides a much stronger evidence that anion channels are involved in the secretion of OA anions from roots under Al stress [69–72]. To date, two families of membrane transporters, the Al-activated malate transporter (ALMT) and the multidrug and toxin compounds extrusion (MATE) families, have been implicated in the secretion of OA anions from plant roots in response to Al. In 2004, Sasaki et al. first isolated the Al-activated OA anion secretion transporter from wheat (i.e., *Al-activated malate transporter 1, TaALMT1*) [73]. Electrophysiological studies show that TaALMT1 functions as a ligand-activated and voltage-dependent anion channel to facilitate malate secretion across the PM of root cells [67, 74, 75]. Following the cloning of the first Al-activated OA anion secretion transporter, *TaALMT1* homologs have been cloned from rape (*Brassica napus*; *BnALMT1 *and *BnALMT2*) [76], *Arabidopsis thaliana *(*AtMALMT1*) [77], and rye (*ScALMT1*) [78]. Osawa and Matsumoto proposed that protein phosphorylation was associated with the Al-activated malate secretion from wheat root apex and that the OA anion-specific channel was possibly a terminal target that responded to Al signal mediated by phosphorylation [79]. Kobayashi et al. observed that the activation of AtALMT1 by Al was inhibited by staurosporine (kinase inhibitor) and calyculin A (phosphatase inhibitor), and that K252a (serine/threonine protein kinase inhibitor) inhibited the Al-dependent malate secretion without reducing gene expression [38]. Ligaba et al. provided evidence indicating that TaALMT1 activity was regulated by protein kinase C-mediated phosphorylation. They observed that TaALMT1 activity was disrupted when the serine residue at position 384 was replaced with an alanine,and concluded that the serine residue needed to be phosphorylated before TaALMT1 was activated by Al. These results suggest that the activation of ALMT1 by Al may involve reversible protein phosphorylation [80]. However, not all ALMT1-type transporters mediate Al-activated OA responses. For example, *ZmALMT1* isolated from maize was suggested to play a role in anion homeostasis and mineral nutrition, and the activity of this protein was independent of extracellular Al [81]. *ZmALMT2* isolated from maize is a root anion transporter that mediates constitutive root malate secretion and may play a role in mineral nutrient acquisition and transport but not Al tolerance [82]. Three ALMT-type transporters isolated from *A. thaliana* were expressed in leaf mesophyll (*AtALMT9*) or guard cells (*AtALMT6* and *AtALMT12*), implicating a primary role in malate homeostasis and guard cell function [83–85]. Recently, Gruber et al. demonstrated that HvALMT1 from barley likely contributed to the homeostasis of OA anions in the cytosol of guard cells and root cells by transporting them out of the cell or into cytosolic vesicles [86, 87].

In 2007, genes encoding citrate transporters that are members of a different transporter family, the MATE family, were isolated from barley (*HvAACT1,* also designated as *HvMATE1*) [88, 89] and sorghum (*SbMATE*) [53]. The MATE family of transporter proteins is a large and diverse group present widely in bacteria, fungi, plants, and mammals. Evidence shows that MATE proteins function as H^+^ or Na^+^ coupled antiporters for numerous substances such as flavonoid, anthocyanins, norfloxacin, ethidium bromide, berberine, acriflavine, nicotine, citrate, and Cd^2+^ [88, 90–92]. Plant MATEs can transport substrates other than citrate, which may also play a role in Al tolerance [88, 90–94]. Recently, MATE homologs involved in Al-activated citrate secretion were isolated from *A. thaliana* (*AtMATE*) [95], rye (*ScFRDL2*) [92], maize (*ZmMATE1*) [96], rice (*OsFRDL4*) [97], and rice bean (*VuMATE*) [98]. All these citrate transporters exhibit varying degrees of constitutive expression (i.e., in the absence of Al) except for *VuMATE*, and their expressions are upregulated by Al treatment except for *HvMATE*. Evidence shows that *OsFRDL4* isolated from rice and *AtMATE *isolated from *A. thaliana* are regulated by a C2H2-type zinc finger transcription factor ART1 and STOP1, respectively [95, 97]. 

In buckwheat, evidence shows that ABA is involved in the secretion of oxalate [99]. ABA activates the anion channel in stomatal guard cells and may play a similar role in the roots [9]. However, no oxalate transporter has been isolated from plants so far.

### 2.2. Internal Concentrations of Organic Acid Anions in Plant Tissues

The effects of Al on OA metabolism have been investigated in some plant species. In an Al tolerant maize single cross, exposure to increasing level of Al led to a strong (over 3-fold) increase in root tip citrate concentration and a significant activation of citrate secretion, which saturated at a rate close to 0.5 nmol citrate h^−1^ root^−1^ occurring at 80 *μ*M Al^3+^ activity, with the half-maximal rate of citrate secretion occurring at about 20 *μ*M Al^3+^ activity [72]. Ligaba et al. demonstrated that Al-treated rape roots had increased *in vitro* activities of citrate synthase (CS, EC 4.1.3.7), malate dehydrogenase (MDH, EC 1.1.1.37) and phospho*enol*pyruvate carboxylase (PEPC, EC 4.1.1.31), and concentrations of citrate and malate, together with decreased respiration rate, concluding that the Al-induced accumulation and subsequent secretion of citrate and malate were associated with both increased biosynthesis and reduced catabolism [59]. In an Al tolerant tree species, *P. facataria*, the Al-induced increases in both secretion and accumulation of citrate were accompanied by increased mitochondrial CS (mCS) activity and enhanced *mCS* expression, indicating that the increased amount of citrate is produced in response to Al [28]. Aluminium treatments resulted in an increase in CS activity and a decrease in aconitase (ACO, EC 4.2.1.3) activity in the root tips of *C. tora*, accompanied by an increase in citrate concentration. However, the activities of NADP-isocitrate dehydrogenase (NADP-IDH, EC 1.1.1.42), MDH, and PEPC were unaffected by Al. It was suggested that Al-regulation of both CS and ACO activities might be responsible for the Al-induced increase in both secretion and accumulation of citrate [100]. Yang et al. reported that CS activity in soybean root apex was increased by 16% when exposed to Al, but the activities of PEPC and NADP-IDH and the concentration of citrate were unaffected. They suggested that the Al-induced increase in CS activity resulted in the increased secretion of citrate [41]. The unchanged concentration of citrate in the Al-treated roots likely reflected the balance between citrate synthesis and secretion in the root apex. In rye, the activity of CS in the root tip increased by 30% when exposed to Al, and the Al-induced increase in the synthesis of citrate appeared to be responsible for the enhanced secretion of citrate from roots [21]. In an Al-tolerant soybean cultivar, mitochondrial MDH and CS activities increased and ACO activity decreased with the increasing of Al concentration and duration of Al treatment. The Al-induced citrate secretion was inhibited by the CS inhibitor suramin and enhanced by the ACO inhibitor fluorocitric acid. Transcript level of the mitochondrial* CS *increased in soybean roots in response to Al, whereas the expression of *ACO* showed no significant difference. These results indicate that Al triggers OA metabolic responses in mitochondria of soybean roots, which support the sustained secretion of citrate [101]. Based on the above results, it is reasonable to believe that altered OA metabolism is involved in the Al-induced secretion of OA anions. However, it is not immediately obvious that simply increasing internal OA will lead to increased secretion, because in any case some transport processes must somewhat be involved in the Al-induced secretion of OA anions. For example, Gaume et al. showed that the Al-tolerant maize cultivar had higher root concentrations and higher root secretion of citrate, malate, and succinate compared with the Al-sensitive one. Increased PEPC activity in root apices after Al exposure partially explained the differences of OA anion concentrations in the roots. However, the increased secretion was not proportional to the OA anion concentrations in the roots. The concentrations of citrate, malate, and succinate in the roots of both cultivars increased by a factor of 2 to 4, whereas the secretion of these anions increased by 2 to 20. They suggested that the secretion of OA anions might be mediated by transporters that are either activated or induced by Al [102]. In a study with two lines of triticale differing in the Al-induced secretion of malate and citrate and in Al tolerance, the concentrations of citrate (root apices and mature root segments) and malate (mature segments only) in roots increased in response to Al, but similar changes were observed in the two lines. The Al-induced changes in *in vitro* activities of CS, PEPC, NAD-MDH, and NADP-IDH were similar in the sensitive and resistant lines in both root apices and mature root segments. These results suggest that the Al-induced secretion of malate and citrate from triticale roots is not regulated by their internal levels in the roots or by the capacity of root tissues to synthesize them [103]. Yang et al. reported that the root concentration of oxalate was poorly related with its secretion among some oxalate accumulators such as *Amaranth *spp., buckwheat, spinach, and tomato [47]. Recently, we observed that Al decreased or did not affect the concentrations of malate and citrate in roots of two citrus species having different tolerance to Al, indicating that the Al-induced secretion of citrate and malate is poorly related to their internal levels in roots [30, 31, 104–106].

Transgenic plants and cells have provided additional evidence that OA metabolism can contribute to the Al-induced secretion of OA anions and Al tolerance [6, 107]. Modulation of OA metabolism enhanced Al tolerance and secretion of citrate and/or malate in transgenic tobacco and papaya (*Carica papaya*) plants overexpressing a *Pseudomonas aeruginosa CS* [108], rape plants [58], and carrot (*Daucus carota*) cells [109] overexpressing a mitochondrial *CS* (*mCS*) from *A. thaliana*, *Nicotiana benthamiana* plants overexpressing an* mCS* from* C. junos *[24], tobacco plants overexpressing a cytosolic *MDH* from *A. thaliana*, an *MDH* from *Escherichia coli* [110] and a *CS* from rice mitochondria [111], alfalfa (*Medicago sativa*) plants overexpressing a alfalfa nodule-enhanced form of *MDH* (*neMDH*) [112], and tobacco plants overexpressing *pyruvate phosphate dikinase* (*PPDK*, EC 2.7.9.1) gene from *Mesembryanthemum crystallinum* [113]. However, Delhaize et al. showed that the expression of a *P. aeruginosa CS* in tobacco did not result in enhanced citrate accumulation or secretion, despite generating transgenic tobacco lines that expressed the CS protein at up to a 100-fold greater level than the previously described CSb lines [40, 108]. They concluded that the activity of the *P. aeruginosa* CS in transgenic tobacco is either sensitive to environmental conditions or that the improvements in Al tolerance and P nutrition observed previously are due to some other variable [40]. 

In an Al-tolerant soybean cultivar, the Al-induced root secretion of citrate increased steadily when exposed to continuous light, and only low citrate secretion was observed under 24 h in the continuous dark. The rate of Al-induced citrate secretion decreased at 6 h after the shoots were excised. The rate of citrate secretion by shoot-excised roots was 3-times lower than that of their respective controls (Al treatment in plants with shoots) during the 6–9 h after 50 *μ*M treatment and 6-times lower during the 9–12 h. These results indicate that the shoots play a role in the Al-induced citrate secretion through providing the carbon source and/or energy for citrate synthesis in the roots [41]. Neumann and Römheld reported that P deficiency strongly increased the concentrations of carboxylic acids in chickpea (*Cicer arietinum*) and white lupin (*Lupinus albus*) roots, but only had small effects on the accumulation of carboxylates in shoots, and suggested that the ability to accumulate carboxylic acids in roots depended on the partitioning of carboxylic acids or related precursors between roots and shoots [114]. Quantification of soybean root enzymes involved in OA metabolism displayed only a 16% increase in CS activity 6 h after Al treatment with no differences in other enzymes; hence citrate may be transported from the shoots to the roots [41]. 

### 2.3. Temperature

Yang et al. observed that the Al-induced secretion of citrate and malate by the roots of *C. grandis* and *C. sinensis* seedlings was inhibited by low temperature, indicating that an energy dependent process may be involved in the Al-induced secretion of OA anions [30]. A similar result has been obtained in Al-tolerant barley [17]. However, the Al-induced secretion of citrate in rye (Pattern II) was decreased by low temperature, but the Al-induced secretion of malate in wheat (Pattern I) was unaffected by low temperature [21]. Recently, Li et al. reported that, in rye, the Al-induced secretion of malate belonged to Pattern I and was not inhibited, while the Al-induced secretion of citrate belonged to Pattern II and was affected by low temperature [60]. Further research is needed to elucidate the mechanism.

### 2.4. Root Plasma Membrane H^+^-ATPase

Since PM H^+^-ATPase plays a critical role in energizing and regulating an array of secondary transporters [115, 116], the modulation of PM H^+^-ATPase activity may be involved in the Al-induced secretion of OA anions. In two soybean cultivars, the Al-induced activity of root PM H^+^-ATPase paralleled the secretion of citrate. The Al-induced increase in PM H^+^-ATPase activity was caused by a transcriptional and translational regulation. Both activity and expression of root PM H^+^-ATPase were higher in the Al-tolerant than in the Al-sensitive cultivar. Aluminum activated the threonine-oriented phosphorylation of PM H^+^-ATPase in a dose- and time-dependent manner. The relationship between the Al-induced secretion of citrate and the activity of PM H^+^-ATPase was further demonstrated by an analysis of PM H^+^-ATPase transgenic *A. thaliana*. When grown on Murashige and Skoog medium containing 30 *μ*M Al, transgenic plants of *A. thaliana* overpressing PM H^+^-ATPase secreted more citrate compared with wild-type *A. thaliana* [117]. Ahn et al. showed that after 4 h *in vivo* treatment with 2.6 *μ*M Al, PM H^+^-ATPase activity and H^+^-transport rate were decreased and *ζ* potential was depolarized in PM vesicles from root tips of Al-sensitive wheat cultivar (ES8) but not of Al-tolerant ET8. They concluded that the Al-induced secretion of malate from wheat roots was accompanied by changes in PM surface potential and activation of H^+^-ATPase [118]. However, the Al-induced changes of root PM H^+^-ATPase activity were not associated with oxalate secretion in two tomato cultivars differing in the ability to secrete oxalate under Al stress [66]. Other studies showed Al inhibited root PM H^+^-ATPase activity in barley [119], squash (*Cucurbita pepo*) [120], and rice bean [121].

### 2.5. Magnesium

Magnesium (Mg) can ameliorate Al toxicity, but the mechanism by which Al alleviates it remains obscure [122, 123]. Long-term secretion of OA anions requires continuous biosynthesis of OAs inside the root cells. In this regard, cytoplasmic Mg^2+^ is pivotal for the activation of many enzymes (e.g., CS, PEPC, IDH, malic enzyme (ME, EC 1.1.1.40), and MDH) involved in OA biosynthesis and degradation [122]. In soybean, micromolar concentration of Mg in the treatment solution alleviated Al toxicity by enhancing citrate biosynthesis and secretion by roots. Increased production and secretion by soybean roots in response to Mg might promote both external and internal detoxification by formation of Al-citrate complexes [123]. In rice bean, Mg could stimulate the Al-induced secretion of citrate from roots thus alleviating the inhibition of root growth by Al. The stimulation of citrate secretion by Mg might result from the restoration of root PM H^+^-ATPase activity by Mg [121]. 

### 2.6. Phosphorus

Phosphorus deficiency is another major factor limiting plant growth in acidic soils [6]. Evidence has shown that Al toxicity can be alleviated by P supply in some plants, including *C. grandis* [30, 31], sorghum [124], maize [102], and *L. bicolor* [125]. There are several authors investigating the effects of P on the Al-induced secretion of OA anions from roots, but the results are somewhat different. Yang et al. showed that the Al-induced secretion of citrate and malate by excised roots from Al-treated *C. grandis* and *C. sinensis *seedlings decreased with increasing P supply, whereas P supply increased or had no effect on the concentrations of both citrate and malate in Al-treated roots [30, 31]. The decreased secretion of OA anions due to P application can be due to the amelioration of Al toxicity by P rather than due to decreased root accumulation of OA anions. In two maize cultivars, the Al-induced increases in root activity of PEPC, root concentrations, and secretion of OA anions were decreased in plants pretreated with higher P concentrations during the 21 days prior to Al treatment [102]. In two cowpea genotypes of contrasting Al tolerance, Al enhanced malate secretion from root apices of both genotypes. Phosphorus deficiency increased the Al-induced secretion of malate by roots only in the Al-tolerant genotype IT89KD-391 [126]. In an Al-tolerant leguminous shrub, *L. bicolor*, the Al-induced secretion of citrate and malate under P sufficiency was less than that under P deficiency [125]. The above results indicate that the enhancement of Al tolerance by P is not associated with an increased secretion of OA anions from roots. However, P-sufficient rape plants displayed more pronounced Al-induced accumulation and secretion of citrate and malate in roots than P-deficient plants. Interestingly, the degree of inhibition of Al-induced root elongation was more or less the same in both P-sufficient and P-deficient plants. It was suggested that the severity of Al toxicity in P-deficient plants was masked by the stimulating effect of P deficiency on root elongation [59]. Using four soybean genotypes differing in P efficiency, Liao et al. investigated the effects of Al and P interactions on OA anion secretion by roots grown in homogeneous and heterogeneous nutrient solutions. In the homogenous solution experiments, P enhanced Al tolerance in four soybean genotypes, but greatly decreased the Al-induced citrate and malate secretion by roots. The two P-efficient genotypes displayed more Al tolerance than the two P-inefficient genotypes under high-P condition, but no significant genotypic difference was found in the secretion of OA anions under both low- and high-P conditions. The secretion of OA anions in a homogenous solution may not reflect the ability of soybean plants to detoxify exogenous Al. At the early stages of the heterogeneous nutrient solution experiment, P greatly increased the rates of the Al-activated citrate and malate secretion from the taproot tips of the four genotypes and the Al tolerance for the two P-efficient genotypes, and the two P-efficient genotypes secreted more malate from the taproot apices under high-P condition. They concluded that, at the early stage of heterogeneous nutrient solution experiment, P might increase the Al-activated secretion of OA anions, thus enhancing Al tolerance [23]. In two soybean cultivars and one rye cultivar, P deficiency did not increase the Al-induced secretion of citrate by roots [60, 127]. In soybean, short-term P deficiency (4 days) followed by Al treatment led to 50% increase in the Al-induced citrate secretion, while longer-term (10 days) P deficiency followed by Al treatment reduced the Al-induced citrate secretion to trace amounts [128]. However, in another study with soybean, Yang et al. showed that application of Al induced a greater citrate secretion rate in the Al-tolerant cultivar than in the Al-sensitive cultivar independently of the P status of the plants [22]. Dong et al. showed that long-term (14 days) P deficiency followed by Al stress (7 h) had no effect on the Al-induced secretion of citrate from soybean roots [127]. This disagreement was attributed to the differences in the plant materials and experimental methods used [128]. Thus, it appears that the influence of P on the Al-induced secretion of OA anions depends on the time of exposure to Al, growth conditions, and plant species or cultivar.

### 2.7. Other Factors

Yang et al. showed that sodium nitroprusside (SNP, a nitric oxide (NO) donor) increased the Al-induced secretion of malate and citrate by excised roots from Al-treated *C. grandis *seedlings and that the stimulatory effects of SNP on the Al-induced secretion of malate and citrate might be involved in the SNP-induced amelioration of Al toxicity [105]. There are several papers reporting that NO regulates K^+^ and Ca^2+^ channels in plants [129, 130]. The stimulation of OA anion secretion by SNP might result from its direct effect on anion channels, because SNP did not enhance the accumulation of malate and citrate in the roots [105]. Chen et al. demonstrated that H_2_S played an ameliorative role in protecting soybean plants against Al toxicity by increasing citrate secretion and citrate transporter gene expression and enhancing the expression of PM H^+^-ATPase [131].

## 3. Genetic Engineering Technology for the Secretion and Biosynthesis of Organic Acid Anions 

A common agricultural practice for acidic soils is to apply lime to raise soil pH. However, the option is not economically feasible for poor farmers, nor is it an effective strategy for alleviating subsoil acidity [132]. A complementary approach to liming practice is to tailor plants to suit acidic soils by identifying and/or developing plants with improved tolerance to Al in acidic soils. The best documented mechanism for plant Al tolerance is the Al-induced secretion of OA anions from roots [6, 9], although it is not the only tolerance mechanism [43, 77]. The production of transgenic plants with an enhanced ability to secrete OA anions appears to be an appealing strategy to produce Al tolerant plants. The two main approaches for increasing OA anion secretion are to increase OA synthesis and to increase OA anion transport across the PM [107].  Table 2 summarizes the attempts to obtain transgenic plants or cells with higher Al tolerance by overexpressing genes involved in the biosynthesis and the secretion of OA anions.

The early attempts to enhance Al tolerance focused on OA synthesis because the genes encoding transporters for OA anions were not cloned at the time. The first influential report of such an approach was from De La Fuente et al. [108] who overexpressed a *P. aeruginosa CS* in tobacco and papaya. Overproduction of citrate was shown to result in Al tolerance in transgenic tobacco and papaya through increasing citrate secretion. Delhaize et al. [40] could not repeat the original study of De La Fuente et al. [108] in the same and other tobacco lines expressing the *CS* from *P. aeruginosa* at higher levels. The authors also observed that *CS*-expressing alfalfa did not show an improved Al tolerance. They concluded that the expression of the *CS* in plants is unlikely to be a robust and easily reproducible strategy for enhancing the Al tolerance of crop and pasture species. Recently, Barone et al. assessed the Al tolerance of 15 transgenic alfalfa overexpressing the *CS* from *P. aeruginosa* by *in vitro* root growth, hydroponics, or soil assay. They deemed that* CS* overexpression could be a useful tool to enhance Al tolerance, but the type of assay used was critical to properly evaluate the transgenic phenotype [133]. Overexpression of mitochondrial *CS *also resulted in increased citrate secretion and enhanced Al tolerance in* A. thaliana* [134], rape [58], *N. benthamiana* [24], and tobacco [111]. In tobacco, overexpression of cytosolic *MDH* genes from *A. thaliana *and from *E. coli *led to increased malate secretion and enhanced Al tolerance [110]. Zhang et al. reported that overexpression of a *C. junos MDH* in tobacco conferred Al tolerance [135]. In alfalfa, overexpression of an alfalfa nodule-enhanced form of *MDH* (*neMDH*) resulted in increased synthesis and secretion of OA anions and enhanced Al tolerance. However, the transgenic alfalfa overexpressing an alfalfa *PEPC* did not show increased accumulation and secretion of OA anions [112]. Trejo-Téllez et al. showed that overexpression of an *M. crystallinum PPDK* in tobacco roots increased the exudation of OA anions, with a concomitant decrease in plant Al accumulation [113]. It should be noted that OA metabolism may not be a limiting factor for Al-induced secretion of OA anions in some plant species because only a small portion of internal OAs is secreted in response to Al [9]. In addition, the Al-induced secretion of OA anions must somewhat be associated with some transport processes [9, 107]. Therefore, the effect that modifying internal OA concentrations has on the level of plant Al tolerance may be small or not observed in some plant species. 

Transgenic plants overexpressing genes encoding transporters for OA anions have been widely studied since the first major gene (*TaALMT1*) was cloned from wheat [73].* TaALMT1* expression in rice, cultured tobacco cells, barley, and *A. thaliana* led to increased Al-activated malate secretion and enhanced Al tolerance for all except for rice [73, 136, 137]. Overexpression of *TaALMT1* in wheat conferred greater Al-activated malate secretion from the roots and improved Al tolerance, which was kept in the T1 and T2 generations [138]. This is the first report of a major food crop being stably transformed for greater Al tolerance. Homologs of *TaALMT1* cloned from *A. thaliana*, rape, barley, and rye [76–78, 87] could also be utilized to increase plant Al tolerance. For example, expression of *BnALMT1* and *BnALMT2* in tobacco cultured cells [76], *HvALMT1* in barley [87], and *AtALMT1* in *A. thaliana *[107] resulted in increased malate secretion and enhanced Al tolerance. Increases in Al tolerance have been achieved by overexpressing citrate transporters. Expression of *SbMATE1* [53], *FRD3* [94], and *ZmMATE1 *[96] in *A. thaliana*, *HvAACT1 *in tobacco [88], and   *VuMATE* in tomato [98] enhanced the secretion of citrate and Al tolerance.

*Arabidopsis thaliana* has one gene encoding for a type I H^+^- pyrophosphatase (AVP1, *Arabidopsis *Vacuolar Pyrophosphatase 1) and another gene encoding for a type II H^+^- pyrophosphatase (AVP2) [139, 142]. Yang et al. reported that overexpression of *AVP1* in *A. thaliana*, tomato, and rice exhibited greater tolerance to Al and higher level of citrate and malate secretion compared with controls and that increased AVP1-dependent H^+^ extrusion appeared to be charge balanced by the enhancement of K uptake and the release of OA from roots [139].

## 4. Concluding Remark 

**Figure 1:**
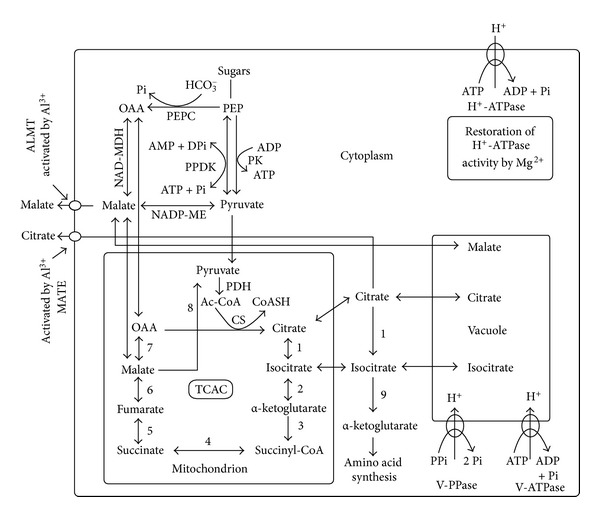
A diagram showing the reactions and processes involved in the accumulation and secretion of organic acid (OA) anions in aluminium- (Al-) treated plants. Ac-CoA: acetyl-CoA: ALMT, Al-activated malate transporter; CS: citrate synthase; DPi: diphosphate; MATE, multidrug and toxin compounds extrusion; NAD-MDH: NAD-malate dehydrogenase; NADP-ME: NADP-malic enzyme; OAA: oxaloacetate; PDH: pyruvate dehydrogenase; PEP: phospho*enol*pyruvate; PEPC: PEP carboxylase; Pi: phosphate; PK: pyruvate kinase; PPDK: pyruvate Pi dikinase; PPi: pyrophosphate; TCAC: tricarboxylic acid cycle; V-ATPase: tonoplast adenosine triphosphatase; V-PPiase, tonoplast pyrophosphatase; 1, aconitase (ACO); 2, NAD-isocitrate dehydrogenase (NAD-IDH); 3, *α*-ketoglutarate dehydrogenase; 4, succinate thiokinase; 5, succinate dehydrogenase; 6, fumarase; 7, NAD-MDH; 8, NAD-malic enzyme (NAD-ME); 9, NADP-IDH (redrawn from Delhaize et al. [14], Anoop et al. [58], Bose et al. [122], Lin et al. [140], and Mariano et al. [141]).

Aluminium toxicity is one of the most deleterious factors for plant growth in acidic soils, which comprise approximately 30% of the world's total land area and over 50% of the world's potential arable lands [1]. In recent years, there has been significant progress in our understanding of the physiological and molecular mechanisms of Al tolerance in higher plants. In particular, the Al-induced secretion of OA anions from roots has been widely studied by many researchers in different plants because it is a major mechanism leading to Al tolerance in higher plants, but the mechanisms which lead to the accumulation and secretion of OA anions are not fully understood (Figure 1). Modulation of OA metabolism and activation of anion channels have been suggested to be involved in the Al-activated secretion of OA anions and transgenic plants or cells overexpressing genes for biosynthesis and secretion of OA anions have displayed increased secretion of OA anions and enhanced Al tolerance (Table 2). It is clear that both ALMT1 proteins from wheat, rye, *A. thaliana*,and rape and MATE proteins from barley and sorghum require Al to activate their function [107]. However, the mechanisms of this activation remain unclear, although evidence shows that the induction of *ALMT1* expression by Al may involve reversible phosphorylation [79, 80, 107] and that *OsFRDL4* and *AtMATE* are regulated by a C2H2-type zinc finger transcription factor ART1 and STOP1, respectively [95, 97]. Although anion channels may play an important role in the Al-induced secretion of oxalate in some plants [47], no oxalate transporter has been isolated from plants so far. Organic acid anions secreted from the roots of P-deficient plants have been shown to mainly result from increased PEPC activity in the shoots [143], but relatively few studies have investigated the roles of shoots in the Al-induced secretion of OA anions. Although some transgenic plants overexpressing *PEPC* or *CS* did not show enhanced accumulation and secretion of OA anions [40, 112], genetically modified plants with higher Al tolerance by overexpressing genes for the secretion and the biosynthesis of OA anions may still be a potentially rewarding area of research in the future. Recent work showed that the Al-activated malate and citrate transporters from the MATE and ALMT families functioned independently to confer Al tolerance of *A. thaliana* [95] and that overexpression of *TaALMT1* in barley, which is very sensitive to Al and does not possess an Al-activated secretion of malate, had an Al-activated secretion of malate with properties similar to those of Al-tolerant wheat and enhanced Al tolerance [136, 137]. Therefore, an additive or a synergistic effect on Al tolerance may be achieved by overexpressing two or more anion transporters regulating the secretion of different OA anions at the same time. If the secretion of OA anions is limited by their supply, genes for OA anion synthesis can be cotransformed with genes for OA anion transport, which may produce transgenic plants with higher levels of Al tolerance. Finally, many transgenic plants with significantly increased Al tolerance will be produced through the collaboration between plant breeders and plant physiologists.

## Figures and Tables

**Table 1:** Characteristics of the aluminum- (Al-) induced secretion of organic acid (OA) anions from roots of different plant species.

Plant species	OA anions secreted	Secretion pattern	Dose response	Temperature sensitivity	Effective inhibitors	References
*Acacia mangium *	Citrate	NA	NA	NA	NA	[28]
*Acacia auriculiformis *	Oxalate, citrate	NA	P	NA	NA	[51]
*Arabidopsis thaliana *	Citrate, malate	NA	NA	NA	NA	[52, 53]
Barley (*Hordeum vulgare*)	Citrate	I	A	P	NIF, A9C	[17]
Buckwheat (*Fagopyrum esculentum*)	Oxalate	I	P	NA	PG	[19, 20, 39, 54]
*Cassia tora *	Citrate	II	P	NA	CHM	[34, 39, 46]
*Citrus grandis *and *Citrus sinensis *	Citrate, malate	I	P	P	CHM (malate), DIDS(1), A9C	[30, 31]
*Citrus junos *	Citrate	II	P	NA	NA	[24]
*Deschampsia flexuosa *	Malate	NA	NA	NA	NA	[55]
*Eucalyptus camaldulensis *	Oxalate, citrate	NA	P	NA	NA	[51]
*Galium saxatile *	Citrate	NA	P	NA	NA	[55]
*Lespedeza bicolor *	Citrate, malate	II	P (malate), A (citrate)	NA	A9C, CHM	[29]
*Leucaena leucocephala *	Citrate	NA	NA	NA	NA	[28]
Maize (*Zea mays*)	Citrate, malate	NA	P	NA	NIF, DIDS(2)	[18, 56]
	Oxalate	NA	A	NA	NA	[8]
*Melaleuca cajuputi *	Oxalate, citrate	NA	P	NA	NA	[51]
*Melaleuca leucadendra *	Oxalate, citrate	NA	P	NA	NA	[51]
Oat (*Avena sativa*)	Citrate, malate	NA	NA	NA	NA	[19]
*Oryza glaberrima *	Citrate	NA	NA	NA	NA	[46]
*Paraserianthes falcataria *	Citrate	NA	NA	NA	NA	[28]
Pea (*Pisum sativum*)	Citrate	NA	NA	NA	NA	[46]
*Polygonum aviculare *and *Polygonum lapathiifolium *	Oxalate	I	NA	NA	PG	[35]
Poplar* (Populus tremula*)	Oxalate, citrate	NA	P	NA	NA	[57]
Radish (*Raphanus sativus*)	Citrate, malate	NA	NA	NA	NA	[19]
Rape (*Brassica napus*)	Citrate, malate	NA	NA	NA	PG	[19, 58, 59]
Rice (*Oryza sativa*)	Citrate	NA	NA	NA	NA	[46]
Rice bean (*Viga umbellata*)	Citrate	II	NA	NA	A9C, NIF, MA, PITC, CHM	[36]
Rye (*Secale cereale*)	Citrate, malate	II	P	P	PP, PITC	[21]
Rye	Citrate, malate	I (malate),	P	P (citrate)	NA	[60]
		II (citrate)		A (malate)		
*Rumex acetosella *	Oxalate	NA	P	NA	NA	[55]
Snapbena (*Phaseolus vulgaris*)	Citrate	NA	NA	NA	NA	[61]
Soybean (*Glycine max*)	Citrate, malate	II	P	NA	NA	[22, 23, 41]
Soybean	Citrate	II	P	NA	A9C, CHM, MA	[62]
Sorghum (*Sorghum bicolor*)	Citrate	NA	NA	NA	NA	[53]
Spinach (*Spinacia oleracea*)	Oxalate	I	P	NA	NA	[35]
*Stylosanthes* spp.	Citrate	II	P	NA	A9C, PITC, PG, NIF, DIDS(1), CHM	[63]
Sunflower (*Helianthus annuus*)	Citrate, malate	NA	NA	NA	NA	[64]
Taro (*Colocasia esculenta*)	Oxalate	NA	P	NA	NA	[65]
Tobacco	Citrate	I	P	NA	NA	[40]
Tomato (*Lycopersicon esculentum*)	Oxalate	I	A	NA	PG	[66]
Triticale (×*Triticosecale *Wittmack)	Citrate, malate	II	P	NA	NA	[26]
*Veronica officinalis *	Citrate	NA	P	NA	NA	[55]
*Viscaria vulgaris *	Oxalate	NA	P	NA	NA	[55]
Wheat (*Triticum aestivum*)	Malate	I	P	A	NIF, DPC, EA, A9C, NPPB, IAA-94	[21, 37, 67, 68]

A: absent; A9C: anthracene-9-carboxylic acid; CHM: cycloheximide; DIDS(1): 4,4′-diisothiocyanatostilbene -2,2′-disulfonic acid; DIDS(2): 4,4′-dinitrostilbene-2,2′-disulfonic acid; DPC: diphenylamine-2-carboxylic acid; EA: ethacrynic acid; IAA-94, (6,7-dichloro-2-cyclopentyl-2, 3-dihydro-2-methyl-oxo-1H-inden-5-yloxy) acetic acid; NA: not applicable; MA: mersalyl acid; NIF: niflumic acid; NPPB: 5-nitro-2-(3-phenylpropylamino)-benzoic acid; P: present; PG: phenylglyoxal; PITC: phenylisothiocyanate;. PP: pyridoxal 5′-phosphate; anion channel inhibitors: A9C, NIF, PG; Citrate carrier inhibitors: MA: PITC, PP; protein synthesis inhibitor: CHM. Two patterns of Al-induced OA anion secretion can be identified on the basis of the timing of secretion. In Pattern I plants, no discernible delay is observed between the addition of Al and the onset of OA anion secretion. In Pattern II plants, OA anion secretion is delayed for several hours after exposure to Al.

**Table 2:** Transgenic plants or cells with higher aluminum- (Al-) tolerance overexpressing genes for the biosynthesis and the secretion of organic acid (OA) anions.

Genes	Origins	Transgenic plants or cells	Increased secretions of OA anions	Al tolerance	References
*Citrate synthase* (*CS*)	*Pseudomonas aeruginosa *	Papaya (*Carica papaya*)	NA	+	[108]
		Tobacco (*Nicotiana tabacum*)	Citrate	+	[108]
		Tobacco	No	No	[40]
		Alfalfa (*Medicago sativa*)	No	No	[40]
		Alfalfa	NA	+	[133]
	*Arabidopsis thaliana *	Rape (*Brassica napus) *	Citrate	+	[58]
		Carrot (*Daucus carota*) cell	Citrate	+	[109]
	*Citrus junos *	*Nicotiana benthamiana *	Citrate	+	[24]
	Carrot	*A. thaliana *	Citrate	+	[134]
	Rice (*Oryza sativa*)	Tobacco	Citrate	+	[111]
*Malate dehydrogenase *(*MDH*)	*C. junos *	Tobacco	NA	+	[135]
	*A. thaliana*, *Escherichia coli *	Tobacco	Malate	+	[110]
Nodule-enhanced form* of MDH (neMDH) *	Alfalfa	Alfalfa	Citrate, oxalate, malate, succinate, acetate	+	[112]
*Phospho*enol*pyruvate carboxylase (PEPC) *	Alfalfa	Alfalfa	No	No	[112]
*Pyruvate phosphate dikinase (PPDK) *	*Mesembryanthemum crystallinum *	Tobacco	Citrate, malate	+	[113]
*TaALMT1 *	Wheat (*Triticum aestivum*)	Tobacco cells	Malate	+	[73]
		Rice	Malate	No	[73]
		Barley	Malate	+	[136, 137]
		Wheat	Malate	+	[138]
		*A. thaliana *	Malate	+	[107]
*AtALMT1 *	*A. thaliana *	*A. thaliana *	Malate		[107]
*BnALMT1* and *BnALMT1 *	Rape	*Tobacco cells *	Malate	+	[76]
*HvALMT1 *	Barley	Barley	Malate	+	[87]
*HvAACT1 *	Barley	Tobacco	Citrate	+	[88]
*SbMATE *	Sorghum (*Sorghum bicolor*)	*A. thaliana *	Citrate	+	[53]
*FRD3 *	*A. thaliana *	*A. thaliana *	Citrate	+	[94]
*ZmMATE1 *	Maize	*A. thaliana *	Citrate	+	[96]
*VuMATE *	*Vigna umbellata *	Tomato	Citrate	+	[98]
Plasma membrane *H* ^+^ *-ATPase *	*A. thaliana *	*A. thaliana *	Citrate	NA	[117]
Type 1 *H* ^+^ *-pyrophosphatase (AVP1) *	*A. thaliana *	*A. thaliana*, tomato, and rice	Malate	+	[139]

NA: not applicable; No: no change in secretion of OA anions or Al tolerance.
